# One-year clinical outcomes of an observational study of static lung preservation at 10° centigrade and semi-elective lung transplantation

**DOI:** 10.1016/j.jhlto.2025.100241

**Published:** 2025-03-05

**Authors:** Christopher M. Bobba, Biplab Saha, Yuriy Stukov, Liam Kugler, Mohammad A. Aladaileh, Olusola Oduntan, William Weir, Jeffrey P. Jacobs, Cynthia Gries, Amir Emtiazjoo, Mindaugas Rackauskas

**Affiliations:** a Division of Thoracic Surgery, Department of Surgery, University of Florida, Gainesville, FL; b Division of Pulmonary, Critical Care & Sleep Medicine, Department of Medicine, University of Florida, Gainesville, FL

**Keywords:** lung preservation, extended lung preservation

## Abstract

**Purpose:**

Lung transplantation (LTx) is performed as soon as donor organs are made available to minimize cold ischemic time. Studies have suggested that an extended static preservation of donor lung allografts at 10°C may offer similar clinical outcomes with the added benefits of surgery being performed as a semi-elective (SE) procedure. The purpose of our study was to compare the clinical outcomes between a cohort of patients who underwent LTx in the traditional fashion and after a period of static preservation at 10°C. Unlike previous studies, we also provide an account of the recipients’ pre-transplant health status.

**Methods:**

This is a prospective, nonrandomized, open label study with propensity matching. Controls were matched using a 2:1 greedy matching algorithm based on LAS within the timeframe of the study (2021–2023), resulting in 15 SE and 30 matched controls. In the SE arm, no LTx was performed between 1800 and 0500 hours. When donor lungs were accepted and the donor cross clamp time occurred between 1800 and 0400 hours, OR time (anesthesia start) was moved to 0600 or later. On arrival, donor lungs were stored at 10°C static preservation. The maximum allowed time between donor cross clamp and recipient anesthesia initiation was 12 hours.

**Results:**

No statistical difference between baseline characteristics was identified between the 2 groups (Table 1). 2/3 of patients were already admitted at the time of transplantation and 15% were on ECMO pre-operatively, indicating a sick patient cohort. Left lung ischemic time (performed second at our institution) was significantly longer in the SE group (722 minutes vs 318 minutes). Post-operative outcomes, including PGD at 72 hours, prolonged MV, ICU and hospital LOS, rejection, re-exploration, and re-admissions were not statistically different between the 2 groups. 1- year survival was 93% for SE patients, and 100% for controls.

**Conclusion:**

One-year outcomes of semi-elective lung transplantation is comparable to traditional transplantation recipients, even in a sick cohort of patients.

## Introduction

Lung transplantation as therapy for end-stage lung disease has evolved tremendously since its first successful completion in 1963.^1^ Although the total number of lung transplantations performed globally continues to rise, the limited availability of donor organs remains a major challenge. Additionally, the inability to preserve donor lungs beyond a limited time has translated into geographic restriction and the ‘emergent’ nature of the transplant surgery. To overcome these barriers, there has been a recent interest in static preservation at 10° centigrade. Studies have shown minimization of cellular damage in both healthy and injured donated lung models at 10° centigrade when compared to standard preservation technique.^2^, ^3^ Extending cold ischemia time (CIT) may lead to many direct and indirect benefits for patients and transplant teams. Longer CIT could allow the team to consider a larger geographical region for potential donors and increase the size of the donor pool for matching donors and recipients. The ability to delay operating room (OR) times and perform the transplantation as a ‘semi-elective’ (SE) procedure in the morning can improve case logistical preparation, team morale, and ultimately reduce the risk of complications.^4^ Possibilities also exist to reduce the cost and carbon footprint by allowing for the use of commercial transportation or external teams in the procurement and transport of donated organs.^5^

Such SE lung transplantation was recently explored in an international trial that suggested non-inferiority compared to standard donor allograft preservation.^6^ Given these results, we pursued a prospective trial of SE lung transplantation in our high acuity patient population. Our research aims to provide additional data on the safety of static preservation at 10° centigrade and provide an example of its feasibility and implementation into broader practice. We also aim to better characterize outcomes based on disease severity and pre-transplant clinical status of the recipients. Therefore, the purpose of this manuscript is to report our initial experience and one-year clinical outcomes from our prospective trial of static lung preservation at 10° centigrade with semi-elective transplantation.

## Methods

### Study design

This is a prospective, observational, open-label single center study in the United States. Our center has consistently performed more than forty lung transplantations every year over the past decade. Fifteen patients underwent delayed transplantation following storage at 10° centigrade in a SE manner described in further detail below. These 15 patients were propensity matched in a 2:1 manner to control patients who underwent transplantation under standard conditions.

### Inclusion and exclusion criteria

Inclusion criteria consisted of lung transplant patients ≥18 years of age who were able to provide informed consent. Exclusion criteria included patients undergoing re-transplantation, multiple organ transplantation, or single lung transplantation. Patients were also excluded if their donor lungs were from a donor >70-years of age or the donor lungs required further evaluation with ex vivo lung perfusion.

All potential eligible transplant recipients were approached for inclusion within the study prior to transplantation. Control patients consisted of those who met inclusion and exclusion criteria who did not receive an organ stored at 10° centigrade. All patients provided written informed consent for inclusion within the study. The University of Florida Institutional Review Board approved the study trial with protocol number IRB202001646.

### Graft preservation techniques

For the SE cohort, if donor cross clamp time occurred between 1800 and 0400 hours, then the recipient operating room time (anesthesia start time) was moved to 0600 or later. The maximum allowed time between donor cross clamp and recipient anesthesia initiation was 12 hours. Lungs were procured in the standard manner including flushing of the donor lungs with a commercially prepared cold low potassium high dextran preservation solution, inflation of the donor lungs with oxygenated air, and storage on ice. On arrival to our institution lungs were then removed from ice and stored at 10° centigrade in a controlled refrigerator. For the control cohort, transplantation proceeded immediately upon donor graft arrival to our institution.

### Transplant surgery and immunosuppression

Lung transplantation at our center is performed on central veno-arterial extracorporeal membrane oxygenation (VA-ECMO) to achieve controlled reperfusion of the graft and minimize ischemia-reperfusion injury. We perform right lung implantation first. We use a clamshell incision for bilateral lung transplantation and a modified clamshell for single lung transplantation. For surgical anastomoses we use a running prolene for the membranous portion of the airway and interrupted polydioxanone (PDS, absorbable) suture for the cartilaginous portion, and use a running Prolene suture for the pulmonary artery and left atrial anastomoses.

We use Basiliximab as an induction agent. Other standard medications at the time of transplantation include methylprednisolone (1gm for bilateral and 500 mg for single lung transplantation) and intravenous mycophenolate mofetil (MMF) 1 gm, given prior to the reperfusion of the first anastomosed lung. We do not use calcineurin inhibitor (CNI) pre-transplantation. CNI is initiated approximately 12 hours after the transplantation if the renal function is stable. The patients receive a second dose of Basiliximab on post-transplant day 4. Tacrolimus is the preferred CNI, generally started sublingually and up-titrated to a target level of 10–15 ng/ml (12–15 for patients 60 years and younger and 10–12 for patients above 60 years). After 6 months, the tacrolimus target is 10–15 and 8–12 for younger and older patients, respectively. The MMF dose is 1000 mg BID unless the recipient develops de novo donor-specific antibody (dnDSA), has risk epitope mismatch (REM), or receives desensitization, in which case, the dose is increased to 1500 BID if tolerated. After an initial higher dose of methylprednisolone, the patients are started on 20 mg of prednisone or equivalent on day 3 and titrated down to 5 mg by 6 months post-transplant. Peri-operative desensitization is routinely performed at our institution for recipients with high cPRA and a positive virtual crossmatch.

### Treatment of rejection

We do not routinely obtain transbronchial lung biopsies (TBBx) until one month post-transplantation. Concerns for acute cellular rejection (ACR) in the early post-transplantation period are often treated empirically with pulse dose methylprednisolone (10mg/kg for 3 days). We follow a specified protocol for the treatment of antibody-mediated rejection (AMR), which includes plasmapheresis, carfilzomib, anti-thymocyte globulin (ATG), and intravenous immunoglobulin (IVIG) if clinically indicated. Beyond one month, we obtain TBBx routinely when there is a clinical concern for rejection, unless clinically prohibitive. Routine screening bronchoscopy with transbronchial biopsies is performed 1, 3, 6 and 12 months after transplantation.

### Outcomes

The primary outcome was the incidence of primary graft dysfunction (PGD) grade 3 at 72 hours after transplantation. PGD grade 3 was defined as the presence of bilateral pulmonary edema on chest x-ray and a P/F ratio <200, consistent with the latest ISHLT Working Group definition on PGD.^7^ Secondary outcomes measured included recipient post-transplant time on ventilator, post-transplant ECMO, airway anastomotic complication, re-exploration in the OR, acute renal failure (requiring temporary dialysis), post-transplant intensive care unit (ICU) length of stay (LOS), post-transplant total hospital LOS, 30-day mortality, 1-year survival, 1-year (or best) post-transplant lung function, and 30-day treatment of acute rejection. Airway anastomotic complication was defined as formation of a bronchopleural fistula or symptomatic dehiscence requiring intervention or symptomatic stenosis requiring intervention.

### Statistical analysis

The SE cohort was propensity matched using a 2:1 greedy nearest neighbor matching algorithm based on the lung allocation score (LAS) within the timeframe of the study (2021–2023), resulting in 30 matched controls. There were a total of 139 lung transplants at our institution within the study timeframe. LAS before and after matching is depicted in Supplemental Table 1. Continuous data is reported as mean (interquartile range [IQR] 25th percentile-75th percentile). Continuous variables were compared using the Mann-Whitney or student t-test. Categorical variables were compared using Chi-squared test. Survival curves were plotted using the Kaplan-Meier method and compared with the log-rank test.

## Results

### Pre-operative and donor characteristics

After 2:1 propensity matching, there were 15 patients in the SE group and 30 matched controls. The demographics, LAS, and indication for transplantation between the SE and control groups were comparable (Table 1). The primary indication for transplantation was interstitial lung disease (ILD) for both groups (73.3% in SE and 50% in control). Importantly, 11/15 (73.3%) of patients in the SE group were already admitted at the time of transplantation, suggesting the high acuity of the patients in the SE cohort. Nine of these patients required high flow nasal cannula (HFNC) and the other two were on veno-venous (VV) extracorporeal membrane oxygenation (ECMO) pre-operatively. 6/15 (40%) of SE patients underwent desensitization for positive virtual crossmatch results. Donor information was also comparable between groups (Supplemental Table 1). There was no difference observed in donor age, cause of death, final PaO2/FiO2 (PF) ratio, or in history of hypertension, diabetes, smoking, or alcohol use.

**Table 1:** Overall and Cohort Characteristics for the Study. Abbreviations: MV, Mechanical Ventilation; BMI, Body Mass Index; LAS, Lung Allocation Score; COPD, Chronic Obstructive Pulmonary Disease; COVID, Coronavirus Disease of 2019; ILD, Interstitial Lung Disease; FEV1, Forced Expiratory Volume in One Second; FVC, Forced Vital Capacity; ECMO, Extracorporeal Membrane Oxygenation; HFNC, High Flow Nasal Cannula; NC, Nasal Cannula; ICU, Intensive Care Unit; LOS, Length of Stay; PGD, Primary Graft Dysfunction

Variable	Overall	Control	Semi-Elective	P-Value

Cohort Size (n)	45	30	15	
Pre-operative Variables				
Age	61 (54, 67.7)	62.8 (55.1, 68.2)	57 (52.5, 62)	0.193
Female Sex	15 (33.3%)	8 (26.7%)	7 (46.7%)	0.314
MV	3 (6.7%)	2 (6.7%)	1 (6.7%)	0.999
BMI	28.7 (25.1, 30.8)	28.5 (24.7, 30.6)	30.1 (25.8, 31)	0.58
Location				0.737
inpatient	30 (66.7%)	19 (63.3%)	11 (73.3%)	
outpatient	15 (33.3%)	11 (36.7%)	4 (26.7%)	
LAS	59.9 (42.1, 67.9)	62.5 (42.3, 68)	59.7 (42, 65.6)	0.665
Diagnosis				0.34
COPD	5 (11.1%)	3 (10%)	2 (13.3%)	
COVID	8 (17.8%)	7 (23.3%)	1 (6.7%)	
ILD	26 (57.8%)	15 (50%)	11 (73.3%)	
Other	6 (13.3%)	5 (16.7%)	1 (6.7%)	
Pre-Op FEV1	1.5 (1.1, 1.8)	1.6 (1.4, 1.9)	1.1 (0.9, 1.8)	0.166
Pre-Op FVC	1.8 (1.6, 2.2)	1.9 (1.7, 2.3)	1.6 (1.1, 2)	0.045
Pre-Op Support				0.698
ECMO	7 (15.6%)	5 (16.7%)	2 (13.3%)	
HFNC	23 (51.1%)	14 (46.7%)	9 (60%)	
NC	15 (33.3%)	11 (36.7%)	4 (26.7%)	
Intra/Post-Op Variables				
Plex Desensitization	12 (26.7%)	6 (20%)	6 (40%)	0.283
1st Lung Ischemic Time	384 (292, 606)	318 (267.2, 383.5)	722 (618.5, 859)	< 0.001
ICU LOS	21 (12.2, 34.8)	20 (11, 29)	29 (14, 53)	0.215
Total LOS	34 (24, 58)	32 (24, 52.8)	55 (24, 69)	0.193
MV >7d (or trach)	14 (31.1%)	8 (26.7%)	6 (40%)	0.569
PGD 3 at 72 h	11 (25%)	7 (24.1%)	4 (26.7%)	0.999
ECMO Post	17 (37.8%)	11 (36.7%)	6 (40%)	0.999
Airway complication	0 (0%)	0 (0%)	0 (0%)	0.999
Re-exploration	10 (22.2%)	5 (16.7%)	5 (33.3%)	0.375
Acute Renal Failure	2 (4.4%)	1 (3.3%)	1 (6.7%)	0.999
30d readmission (unplanned)	9 (20.5%)	5 (16.7%)	4 (28.6%)	0.61
Acute Rejection Treatment	13 (28.9%)	8 (26.7%)	5 (33.3%)	0.907
Best post FEV1	2.3 (1.8, 2.9)	2.5 (1.9, 3)	1.9 (1.7, 2.6)	0.104
FEV1 Change	49.7 (17.8, 84)	49.7 (15.5, 87.3)	50.5 (32, 70.3)	0.826
FVC Change	41.8 (23.6, 76.5)	37.1 (22.9, 67.4)	57.4 (26.1, 78.7)	0.465
1-year Survival (%)	97.8	100	93.3	

### Intra-operative and posttransplant characteristics

**Figure 1:**
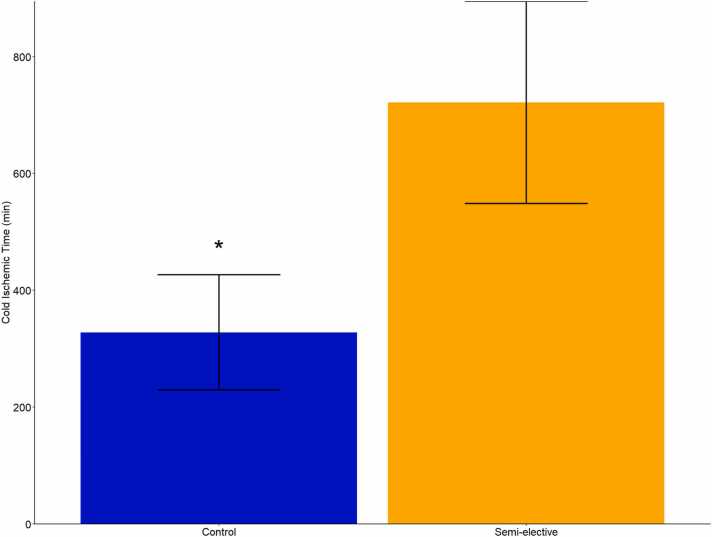
Average cold ischemic time for each group. Bar demonstrates mean with error bars expressing standard deviation. * p<0.05.

**Figure 2:**
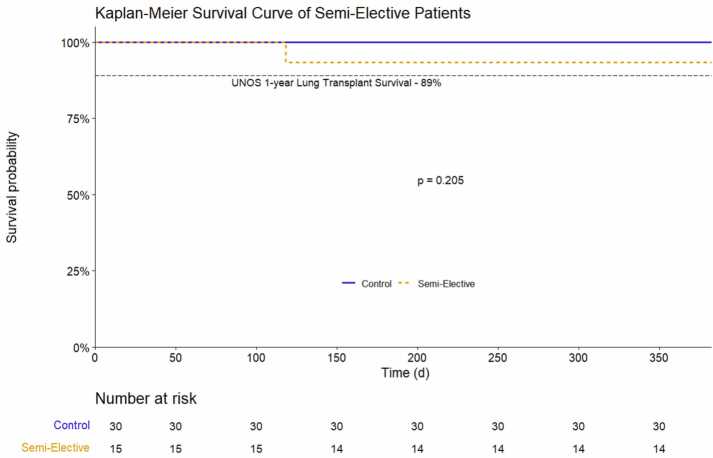
Kaplan-Meier plot of 1-year survival for control and semi-elective cohorts. P-value derived from log-rank test comparing two groups.

Left lung ischemic time (performed second at our institution) was significantly longer in the SE group (722 minutes vs 318 minutes, Figure 1). The overall incidence of PGD 3 at 72 hours was 25%. There was no difference in this primary outcome of the incidence of PGD grade 3 at 72 hours after transplantation between the SE cohort (4/15=26.7%) versus the control cohort (7/30=24.1%). No statistically significant differences were observed in recipient post-transplant time on ventilator, post-transplant ECMO, post-transplant ICU LOS, post-transplant hospital LOS, 30-day mortality, and 1-year survival (Table 1, Figure 2). Total rate of posttransplant ECMO for both groups was 37.8%. The average total hospital stay for all patients was 34 days. Overall survival for all patients at 1 year was 97.8%. There we no deaths in the control cohort, and 1 death in the SE cohort. There was no difference in 1-year survival between control and SE patients (log-rank test, p=0.205, Figure 1). Readmission and 30-day treatment of acute rejection were not different between the SE cohort and the control patients. No differences were observed between other intra-operative and post-transplant characteristics (Table 1).

## Discussion

Our study further extends the knowledge regarding SE lung transplantation and adds to the existing medical literature. Overall, in our high-acuity patient population, we did not observe any important differences in outcome between lungs transplanted in the standard fashion and those transplanted following preservation at 10°C and SE transplantation.

The optimal temperature for the preservation of donor lungs is debatable. The current standard technique employs antegrade and retrograde flushing of the donor lungs with a commercially prepared cold low potassium high dextran preservation solution, inflation of the donor lungs with oxygenated air, and subsequent storage on ice to maintain a temperature of roughly 0°C to 4°C, though some recent studies have indicated a wide variation in regional organ temperature when transported using this method.^8^, ^9^ Despite many modifications in the surgical approach and other aspects of lung transplantation over the last four decades, the preservation technique with ice has remained relatively constant.

Traditional ice preservation is cheap and is considered the gold standard. This technique is often performed using the triple-bag technique, where the allograft is inflated and packaged into 3 plastic bags, followed by placement into an ice cooler for transport. While ice transportation attempts to maintain mean temperatures of around 4°C, it is unable to provide uniform surface temperature in all areas of the lungs. In fact, the regional temperature differences could be substantial (0.26°C to 10.6°C), raising the possibilities of lung freezing and contact damage as well as ischemic injury.^8^ Preservation on ice results in the denaturation of the cellular enzymes, causing a shift towards anaerobic metabolism, lactate production, ATP depletion, cellular acidosis, and free radical injury.^10^ Additionally, dysfunctional cell membrane proteins result in sodium and calcium influx, leading to the inability to maintain intracellular ionic composition, causing mitochondrial damage and precipitating apoptosis.^11^, ^12^ Mitochondrial damage during prolonged ice preservation is thought to be the driving factor behind organ dysfunction. Preclinical studies performed as far back as 30 years demonstrated better lung preservation at 10° centigrade compared to 4° centigrade.^9^, ^13^, ^14^ A recent study using a large animal model revealed better physiologic parameters as well as preserved mitochondrial function when the lungs were preserved at 10° centigrade compared to 4° centigrade.^2^ These data compel researchers to find better ways to control temperature and manage ischemia in the donor lung.

The purpose of hypothermic static cold storage is to slow down the cellular metabolism of the donor lungs and extend the CIT while still allowing for safe implantation with excellent outcomes. Some previous data have shown that a longer CIT may be associated with worse post-transplant outcomes; and therefore, most lung transplant centers practice a CIT cut off of 6–8 hours.^15^ A recent registry analysis indicated a median allograft ischemic time of 5.5 hours for bilateral lung transplantation.^16^ This limitation in CIT creates a bottleneck in transplantation by limiting the geographical regions in which lungs may be available for procurement and therefore limiting the potential donor pool for matched recipients. Additionally, this limitation in CIT compels lung transplantation to be an urgent procedure, often occurring in the middle of the night and in a continuous fashion. Several studies, including a meta-analysis, have demonstrated worse mortality among patients who have undergone surgery in the afterhours or at night time compared to surgery performed during the day, even after adjusting for patient and surgical characteristics.^17^ With this forced urgency for lung transplantation, there is a higher risk of post-transplant adverse events and reduced 5-year overall survival and Bronchiolitis obliterans syndrome (BOS)-free survival.^18^

PGD is a serious and potentially life-threatening complication following lung transplantation. Severe PGD (grade 3) is associated with reduced chronic lung allograft dysfunction (CLAD) -free survival, especially when it persists 72 hours after transplantation. Lung damage during ice preservation has been theorized as a potential contributor to the development of PGD. Based on preclinical studies, preservation of the lungs with 10° centigrade has been postulated to protect against the development of PGD.^6^ Our study demonstrated an overall high incidence of PGD 3 at 72 hours (24.5%), with no difference observed between SE and control groups. This rate is higher than previously reported in studies evaluating prolonged cold static preservation.^7^ We believe this finding is because of the overall high acuity of illness pre-transplantation. 38/54 (70%) of our patients were either receiving oxygen via HFNC (78.9%) or were on ECMO (21%). Importantly, consistent with previous publications, our study showed a similar incidence of PGD 3 in the SE cohort and control patients.^6^, ^19^

In terms of secondary outcome measures, no significant differences were observed between the two cohorts regarding the short-term peri-transplant outcomes. Of note, no difference was found in the incidence of acute renal failure between the groups, which tends to portend poor outcomes.^20^ Despite having a much longer CIT, there were no deleterious effects regarding post-transplant coagulation abnormalities or bleeding requiring chest re-exploration in the SE group. The ICU LOS, overall hospital LOS, and readmissions were also comparable between the SE cohort and the control patients. Acute rejection after lung transplantation is associated with worse survival and development of CLAD. Acute rejection is common following transplantation, especially in the first year.^21^ The number of patients requiring treatment for acute rejection was not different between the SE cohort and the control patients, further emphasizing the safety of the SE procedures.

Two prior studies have investigated transplantation under a similar protocol.^6^, ^19^ The demographics of our recipients are comparable to these studies. Additionally, the total ischemia time, use of post-transplant ECMO, and incidence of rejection are comparable. However, these previously published studies have not specified the number of patients who received lung transplantation as an inpatient rather as a planned outpatient admission. The acuity of our patients further adds to this accumulating breadth of data supporting preservation at 10° centigrade.

There are several limitations to our study. Though this was a prospective trial, it was non-randomized and observational in nature. Our statistical analysis did not include a number needed to treat (NNT) analysis because the intention of our study was as a “proof of concept” study, to provide baseline information regarding safety, and therefore generate data to support a larger future randomized trial. Caution should be taken before any causal inference is made from these results. Also, for simplicity, we performed matching to controls using the surrogate LAS (which takes in to account pre-operative characteristics and predicted post-transplant survival) rather than specific pre-operative characteristics or comorbidities. Additionally, lungs were procured and transported on ice before being stored in a 10° centigrade refrigerator. Maximization of the advantage of 10° centigrade preservation would include immediate storage of the organ at 10° centigrade upon procurement and subsequent transportation at 10° centigrade. Future studies should therefore work toward the development of a transportable system allowing storage and transport at 10° centigrade.

## Conclusions

The SE approach to lung transplantation appears to be safe. Further, our unique and high-acuity patient cohort provided additional data suggesting that SE transplantation may still be a viable option in sicker, more tenuous patients. Our peri-transplant and short-term outcomes in SE patients, including one-year survival, are comparable to our outcomes in patients undergoing standard ‘emergent’ lung transplantation. Adopting the SE technique may make the lung transplantation procedure safer, expand the door pool, reduce cost, and attract a new generation of physicians to the field of organ transplantation. Future randomized studies of this technique should further study the safety non-inferiority of this transplantation technique.

## Declaration of Competing Interest

The authors declare that they have no known competing financial interests or personal relationships that could have appeared to influence the work reported in this paper.

## Appendix A Supplemental material

Supplemental Table 1. Donor characteristics for each cohort of the study. Abbreviations: CVA, cerebrovascular accident; CNS, central nervous system

## Footnotes

Appendix A Supplemental data associated with this article can be found in the online version at doi:10.1016/j.jhlto.2025.100241.
