# Supplementary material for: One-year clinical outcomes of an observational study of static lung preservation at 10° centigrade and semi-elective lung transplantation
Source: JHLT Open. 2025 Mar 5;9:100241. doi: 10.1016/j.jhlto.2025.100241 (PMC12141638; doi:10.1016/j.jhlto.2025.100241)
Supplement: Supplementary file 1 — Supplemental Table 1. Donor characteristics for each cohort of the study. Abbreviations: CVA, cerebrovascular accident; CNS, central nervous system [file mmc1.pdf]

Variable	Overall	Control	Semi-Elective	P-Value
Cohort Size (n)	45	30	15	
**Donor Variables**				
Age	28 (22, 41)	27 (21, 36)	37 (25,43)	0.242
Diabetes	3 (6.7%)	2 (6.7%)	1 (6.7%)	0.999
Hypertension	11 (24.4%)	7 (23.3%)	4 (26.7%)	0.999
Smoking, >20 pack-year	4 (9.1%)	2 (6.9%)	2 (13.3%)	0.88
Heavy Alcohol Use	5 (11.6%)	3 (10.3%)	2 (14.3%)	0.999
Cause of Death				0.264
Anoxia	12 (26.7%)	6 (20%)	6 (40%)	
CVA	12 (26.7%)	7 (23.3%)	5 (33.3%)	
Head Trauma	20 (44.4%)	16 (53.3%)	4 (26.7%)	
CNS Tumor	1 (2.2%)	1 (3.3%)	0 (0%)	
PF Ratio	507 (445, 570)	505 (434, 571)	510 (466, 556)	0.613
