# Supplementary figures and images for: One-year clinical outcomes of an observational study of static lung preservation at 10° centigrade and semi-elective lung transplantation
Source: JHLT Open. 2025 Mar 5;9:100241. doi: 10.1016/j.jhlto.2025.100241 (PMC12141638; doi:10.1016/j.jhlto.2025.100241)

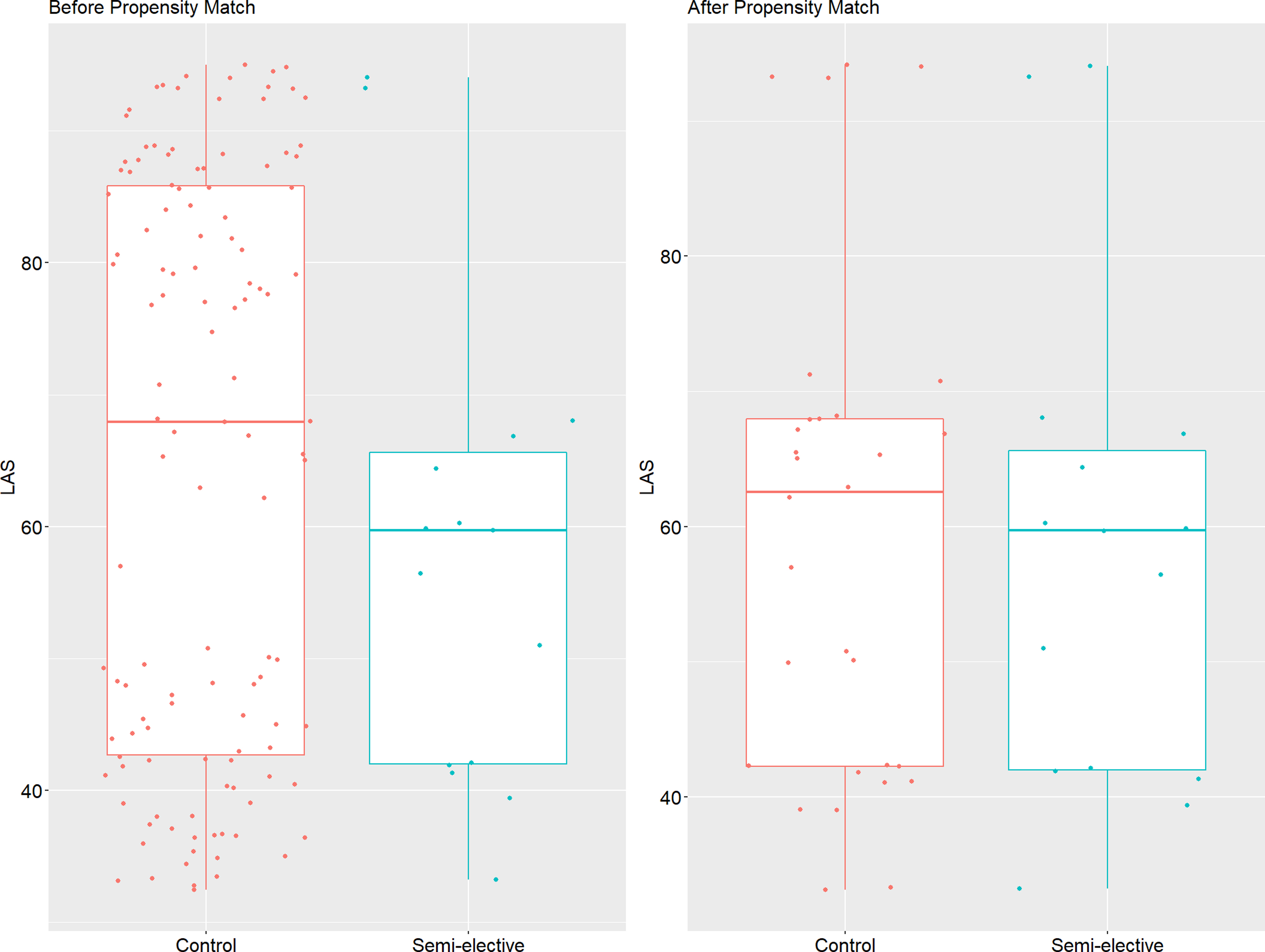

Supplement: Supplementary file 2 — Supplemental material [file mmc2.jpg]
